# Effectiveness of Medical Treatment of Cushing’s Disease: A Systematic Review and Meta-Analysis

**DOI:** 10.3389/fendo.2021.732240

**Published:** 2021-09-17

**Authors:** Julia Simões Corrêa Galendi, Afonso Nogueira Simões Correa Neto, Michelle Demetres, Cesar Luiz Boguszewski, Vania dos Santos Nunes Nogueira

**Affiliations:** ^1^University of Cologne, Faculty of Medicine and University Hospital of Cologne, Institute of Health Economics and Clinical Epidemiology, Cologne, Germany; ^2^Medical School, Federal University of Minas Gerais, Belo Horizonte, Brazil; ^3^Samuel J. Wood Library & C.V. Starr Biomedical Information Center, Weill Cornell Medicine, New York, NY, United States; ^4^Department of Internal Medicine, Endocrine Division (SEMPR), Federal University of Parana, Curitiba, Brazil; ^5^Department of Internal Medicine, São Paulo State University/UNESP, Medical School, Botucatu, São Paulo, Brazil

**Keywords:** Cushing’s disease, pasireotide (SOM230), cabergoline, ketoconazole, metyrapone, systematic literature review, meta-analysis

## Abstract

**Objective:**

The objective of this systematic review was to evaluate the effectiveness and safety of pasireotide, cabergoline, ketoconazole, levoketoconazole, metyrapone, osilodrostat, and temozolomide for the treatment of Cushing’s disease (CD).

**Methods:**

The primary outcomes were the proportion of CD control, adverse events (AE), and reduction of urinary free cortisol. Search strategies were applied to Embase, Medline, and CENTRAL. Independent reviewers assessed the study eligibility, extracted data, and evaluated risk of bias. Standardized mean difference was calculated with 95% confidence interval (CI) for continuous data (*i.e*., pre- and post-intervention). Random meta-analyses for the proportion of CD control and AE were conducted.

**Results:**

Twenty-nine controlled and non-controlled studies were included. No study with temozolomide and levoketoconazole and one study with osilodrostat fulfilled the inclusion criteria. The meta-analyses of proportion of CD control was 35% for cabergoline (95% CI: 27–43%, six studies, 141 participants), 44% for pasireotide (95% CI: 25–35%, eight studies, 522 participants), 41% for ketoconazole (95% CI: 36–46%, six studies, 450 participants), 66% for metyrapone (95% CI: 46–87%, four studies, 66 participants), and of 66.4% for osilodrostat (95% CI: 57.9, 74.3, 97 participants, one study). One study compared two different treatments (cabergoline *vs*. ketoconazole), and no statistical difference was observed in CD control (RR: 0.53, 95% CI: 0.15 to 1.87, 14 participants, very low certainty of evidence). The most frequent AE associated with pasireotide was hyperglycemia, dizziness and nausea with cabergoline and metyrapone, and elevated transaminases with ketoconazole.

**Conclusion:**

The superiority of one drug over another could not be determined due to lack of controlled studies, but the proportion of disease control identified in our meta-analysis may support clinical decision. New therapeutic options should be investigated due to the limited efficacy and tolerability of the currently available medical treatment for patients with Cushing’s disease.

**Systematic Review Registration:**

https://www.crd.york.ac.uk/prospero/display_record.php?ID=CRD42020205567, identifier CRD42020205567.

## Introduction

Cushing’s disease (CD) results from an ACTH-secreting pituitary adenoma and is the main cause of endogenous hypercortisolism in adults. The incidence of CD is 1.2 to 2.4 patients per million each year (1). The first-line treatment for CD is transsphenoidal surgery (TSS), which can lead to disease control in 68 to 98% of patients (2). Late recurrence of the disease after TSS has been reported to occur in 15 to 66% of patients at 5 to 10 years after surgery, which was considered successful (3, 4). Patients who underwent TSS without success and those with contraindications for surgical treatment might benefit from medical treatment (5). Medical treatment is also recommended to control severe hypercortisolism before surgery or while awaiting the effects of radiotherapy treatment (6, 7).

Three main categories of medical treatment can be identified according to the mechanism of action: pituitary-, adrenal-, and glucocorticoid receptor (GR)-directed drugs (8). Mifepristone is the main GR-directed drug, and although it improves the clinical burden of chronic hypercortisolism, it does not affect cortisol secretion (9).

Pituitary-directed drugs, namely, pasireotide, cabergoline, and temozolomide, target the corticotroph pituitary tumor directly. Pasireotide is a somatostatin analogue with high affinity for the SST5 receptor that decreases ACTH production. Two formulations of pasireotide are available, subcutaneously administered twice daily (600 and 900 mcg), and have a long-acting release formulation, requiring a single intramuscular administration every 4 weeks (10 and 30 mg). Disease control might be achieved in 20 to 60% of patients with CD who remained uncontrolled after surgery (10, 11).

Cabergoline is a long-acting dopamine agonist that might inhibit ACTH secretion by acting on dopamine receptor subtype 2. The control of hypercortisolism might be achieved in up to 40% of patients (12), while others have found cabergoline to be of little value in the therapy of CD (13).

Adrenal-directed drugs induce a decrease of cortisol secretion through the inhibition of steroidogenesis. Ketoconazole is an imidazole derivative that inhibits several enzymes, such as 17,20-lyase and 11β-hydroxylase. Ketoconazole induces control in 30 to 80% of patients with Cushing’s syndrome (14). Additionally, metyrapone is an adrenal enzyme blocker, mainly acting on 11b-hydroxylase, and has been extensively studied for the treatment of Cushing’s syndrome, showing an average control rate of 75.9% (14).

New adrenal-directed drugs have been recently developed, such as levoketoconazole and osilodrostat. Levoketoconazole is the cis-2S,4R steroisomer of the classical racemic ketoconazole, showing a similar enzymatic inhibitory profile and found to be more potent in experimental models (15). Osilodrostat potently inhibits the adrenal enzymes aldosterone synthase and 11b-hydroxylase, therefore inducing a decrease in glucocorticoid and mineralocorticoid production and secretion (16). Moreover, the efficacy of retinoic acid, which acts on the proopiomelanocortin gene transcription and inhibits corticotropinoma development, was assessed in a small cohort (17).

There is still uncertainty on the effectiveness and safety of the alternative medications to patients with CD. Therefore, the aim of this systematic review was to assess the effectiveness and safety of medical treatment for patients with uncontrolled CD who underwent TSS or who had contraindications to surgery as first-line treatment, with at least 6 months of follow-up.

## Materials and Methods

This systematic review is reported according to the PRISMA statement (18) and was registered at PROSPERO (CRD42020205567).

### Eligibility Criteria

We included randomized and non-randomized controlled trials and non-controlled studies that were in accordance with the criteria below.

#### Patients

Adults with diagnosis of CD, who did not fulfill control criteria after TSS, who presented a recurrence of Cushing’s after a postoperative period of eucortisolism, or who had contraindications for surgery as first-line treatment were included in the study. We considered as having CD patients with clinical manifestations of the disease associated with at least two positive screening tests for hypercortisolism, a baseline plasma ACTH level >20 pg/ml, and confirmed ACTH-secreting pituitary adenoma after surgery. For symptomatic patients who did not undergo surgery or whose tumor could not be identified after surgery, we considered as diagnostic criteria a bilateral inferior petrosal sinus catheterization or a magnetic resonance image evidencing a pituitary adenoma >6 mm (19).

#### Intervention and Comparison

Monotherapy with pasireotide, cabergoline, ketoconazole, levoketoconazole, metyrapone, osilodrostat, and temozolomide was considered.

#### Outcomes

The primary outcomes were as follows: proportion of disease control as defined by the authors and proportion of adverse events (AE), with the latter reported according to the Common Terminology Criteria for Adverse Events (20). The secondary outcomes were improvement of urinary free cortisol (UFC) and comorbidities associated to CD (*i*.*e*., weight loss, improvement of diabetes mellitus, waist circumference, hypertension, and cholesterol). Serious adverse events (SAE) were those that resulted in death, hospitalization, or prolongation of existing hospitalization, a persistent or significant incapacity, substantial disruption of the ability to conduct normal life functions, or a congenital anomaly (21).

### Exclusion Criteria

To minimize the risk of selection bias, at least 10 patients had to be included in the studies. In case of overlapping populations, the article with the largest sample and more complete reporting of data was included.

### Search Strategy

Three general search strategies were developed for the main electronic health databases: Embase (1980–August 20, 2020), PubMed/Medline (1966–August 20, 2020), and Cochrane Collaboration Controlled Trials Register (1982–August 20, 2020). A second search on all databases was conducted on January 16, 2021. The strategies for PubMed and Embase were reviewed by a medical librarian (MD) using the PRESS 2015 Evidence-Based Checklist tool (22). The search strategies included the following descriptors and synonyms: Cushing’s disease, cabergoline, pasireotide and ketoconazole, osilodrostat, levoketoconazole, metyrapone, and temozolomide. The complete search strategy for Pubmed/Medline is provided in the supplementary material (**Supplementary Tables S1A**, **S2A**). To search for gray literature, we checked for ongoing studies on ClinicalTrials.gov, references of articles selected for full reading, and annals of congress. There was no language restriction.

### Selection of Studies

Two reviewers (JSCG and VSNN) independently reviewed the titles and abstracts. Potentially eligible studies were selected for full reading, to be assessed for adequacy to the PICO previously established. In case of disagreement, a consensus meeting was made.

### Data Extraction and Risk of Bias of the Included Studies

Two reviewers (JSCG and ANSCN) used a standardized form to independently extract relevant data of the included studies and to assess the risk of bias of the included studies. In case of disagreement, a consensus meeting was made. To assess the risk of bias of the included studies, the critical appraisal tool from Joanna Briggs’s Institute was adapted to check the included studies with regard to the following aspects: (i) clear inclusion criteria, (ii) diagnostic criteria stated, (iii) description of valid biochemical assays to measure hypercortisolism, (iv) consecutive and complete inclusion of participants, (v) complete reporting of baseline information, (vi) complete reporting of outcomes, (vii) complete reporting of demographics of the site, and (viii) appropriate statistical analysis. For each aspect, we assigned yes, no, or unclear (23).

### Synthesis and Analysis of Data

Homogeneous endpoints in at least two studies were plotted in meta-analyses using the Stata Statistical Software 16 (Stata Statistical Software: Release 16, College Station, TX, StataCorp LLC, USA). Proportional meta-analyses were performed for dichotomous data. We used the updated command *metaprop_one* and fit the logistic-normal random-effects model to the data (20). Continuous data were expressed as means and standard deviation (SD), and the pre- and post-intervention standardized mean difference (SMD) were calculated with respective 95% confidence interval (CI).

Inconsistencies between the results of the studies included were ascertained by a visual inspection of forest plots and by applying the Higgins statistic (*I* ^2^) and the chi-square test (*χ* ^2^). Moderate heterogeneity was ascertained if *I* ^2^ >35%. For *χ* ^2^, statistic heterogeneity was considered if *p <*0.10 (21). In order to explore the potential sources of heterogeneity, meta-regression was performed using logit transformed outcomes and logit transformed with study SD. The study sample size, study design (*i*.*e*., randomized, prospective), mean age of the study participants, and doses of the intervention were considered as potential explanatory variables. The Knapp–Hartung correction was used to calculate the significance of the meta-regression coefficients (24).

Prediction interval (PI) was calculated for the random-effect meta-analysis, if *χ* ^2^ *p <*0.1 or *I* ^2^ >35% and more than five studies. PI predicts the possible treatment effect in an individual study setting, whereas the random effect meta-analysis summarizes the average effect across the studies (25). Because the potential treatment effect when applied within an individual study setting may differ from the average effect, the PI provides interesting insights for clinical practice (25).

### Quality of the Evidence

For the outcomes from controlled studies, the quality of evidence for estimating the effect of intervention was generated in accordance with the Grading of Recommendations Assessment, Development, and Evaluation Working Group (26).

## Results

### Study Selection

**Figure 1:**
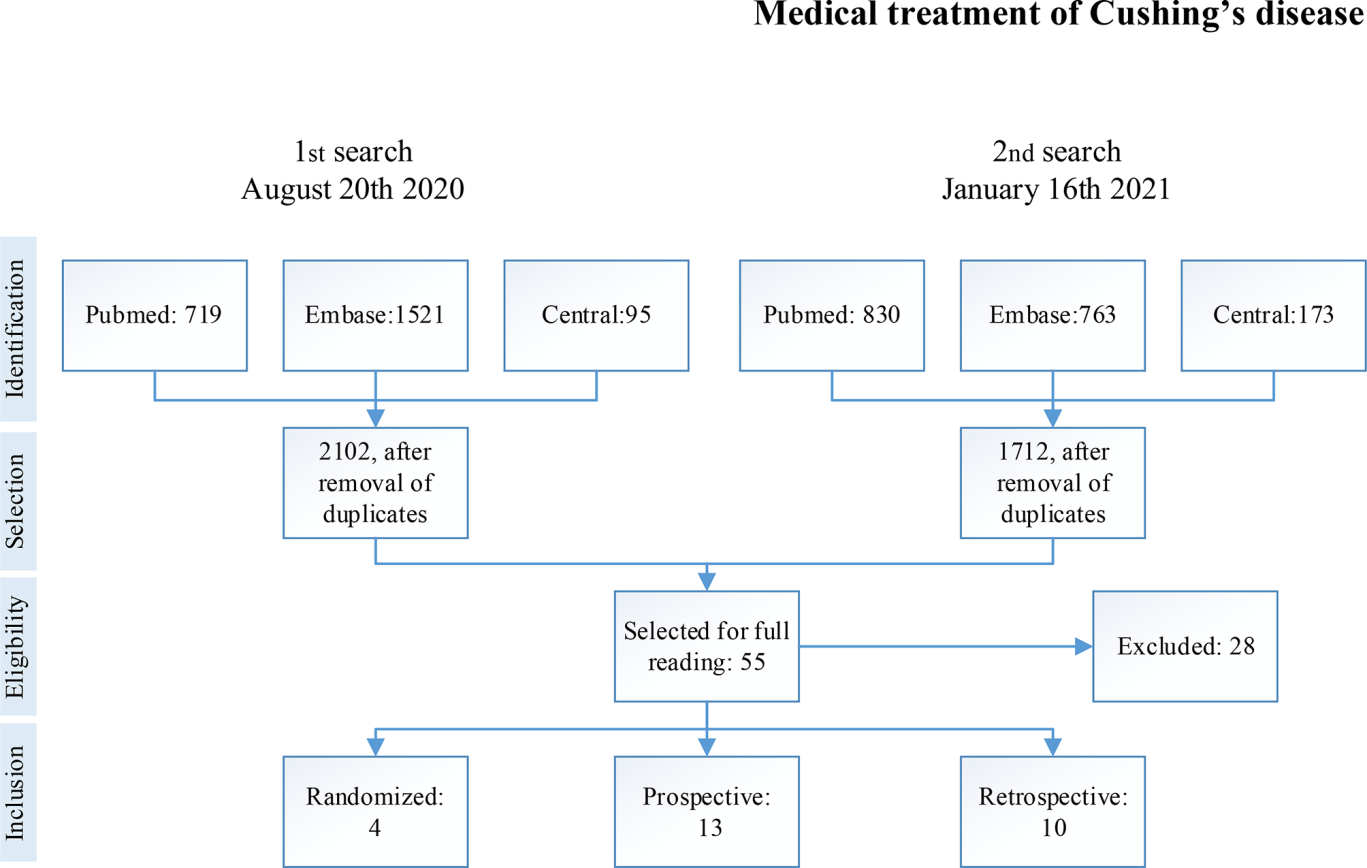
Process of selection of studies.

The search strategies resulted in 2,102 and 1,712 articles after duplicates were removed using the Endnote software. We selected 55 articles for full reading, of which 27 (1,405 patients) were included. Although we set out to include patients with CD only, we included for full reading studies that sampled other etiologies of Cushing’s syndrome and tried contacting authors to retrieve the data for CD only. Among the excluded studies, nine studies had overlapping population with other already included studies (27–35), seven studies were cohorts of less than 10 patients (36–42), three did not match the study population (43–45), six did not comply with the outcomes (46–51), and three assessed outcomes before six months of follow-up (13, 52, 53). The selection process is summarized in **Figure 1**.

### Characteristics of the Included Studies

The characteristics of the included studies are reported in the supplementary material (**Supplementary Table S3A**). We included eight studies on pasireotide (518 patients) (10, 11, 54–59), six studies on cabergoline (139 patients) (12, 60–64), 10 studies on ketoconazole (559 patients) (60, 65–73), five studies on metyrapone (160 patients) (66, 69, 74–76), and one study on osilodrostat (36 patients) (77).

There were four randomized controlled studies (10, 54, 60, 77) and 23 single-arm studies, from which 13 were prospective (11, 12, 55–58, 61, 62, 65, 67, 68, 73, 75) and 10 were retrospective (59, 63, 64, 66, 69–72, 74, 76). To confirm the pituitary origin of Cushing’s syndrome, the selected studies considered dynamic tests, in addition to the criteria pre-established in our review protocol. Four studies considered the 8-mg/day high-dose dexamethasone suppression test (12, 63, 71, 72), four studies considered the corticotropin-releasing-hormone test (10, 68, 73, 75), and 10 considered both (59–61, 64–67, 69, 70, 74).

Among the three randomized controlled studies included, only one compared two different medications. Data plotted in the meta-analysis refers to the 6-month follow-up of the study by Barbot et al., in which both cabergoline and ketoconazole were used as monotherapy. No statistical difference was observed in the CD control (relative risk: 0.53, 95% CI: 0.15 to 1.87, 14 participants, very low certainty of evidence). The quality of evidence was rated down due to imprecision (*i*.*e*., wide CI and no achievement of optimal information size) and high risk of selection bias (60). Two randomized studies compared different dosages of pasireotide (*i*.*e*., Colao et al. compared 600 *vs*. 900 mcg and Lacroix et al. compared 10 *vs*. 30 mg long-acting release) (10, 54).

### Risk of Bias of the Included Studies

The description of inclusion criteria was adequate in all the included studies. In 65% of the studies, it was unclear if the inclusion of patients was consecutive and complete. Reporting was unsatisfactory in 24% of the included studies regarding baseline information and in 48% regarding outcomes. Statistical analysis was considered inappropriate in 20% of the studies. **Table 1** shows the risk of bias of the included studies.

**Table 1:** Risk of bias of the included studies.

Author	Year	1. Clear inclusion criteria	2. Diagnostic criteria stated	3. Valid biochemical assay to measure hypercortisolism	4. Consecutive and complete inclusion of participants	5. Complete reporting of baseline information	6. Complete reporting of outcomes	7. Complete reporting of site demographics	8. Appropriate statistical analysis	Overall risk of bias^a^
Barbot et al. (60)	2014	✅	✅	✅	✅	✅	✅	✅	✅	Low risk
Colao et al. (10)	2012	✅	✅	✅	✅	✅	✅	✅	✅	Low risk
Lacroix et al. (54)	2018	✅	✅	✅	✅	✅	✅	✅	✅	Low risk
Albani et al. (55)	2018	✅	✅	✅	⚠	❌	✅	✅	✅	High risk
Barbot et al. (11)	2018	✅	✅	✅	✅	❌	✅	✅	❌	Some concerns
Boscaro et al. (56)	2014	✅	✅	✅	⚠	✅	✅	✅	✅	Some concerns
Fleseriu et al. (57) (pasireotide)	2018	✅	✅	✅	⚠	✅	❌	✅	✅	High risk
Pivonello et al. (58)	2019	✅	✅	✅	⚠	✅	✅	✅	✅	Some concerns
Vilar et al. (61)	2010	✅	✅	✅	⚠	✅	❌	✅	✅	High risk
Lila et al. (62)	2010	✅	✅	❌	⚠	✅	❌	✅	✅	High risk
Pivonello et al. (12)	2009	✅	✅	✅	⚠	✅	✅	✅	✅	Some concerns
Castinetti et al. (72)	2008	✅	✅	⚠	✅	❌	❌	✅	❌	High risk
Castinetti et al. (71)	2014	✅	✅	⚠	⚠	✅	✅	✅	✅	Some concerns
Invitti et al. (70)	1999	✅	✅	✅	⚠	❌	❌	✅	❌	High risk
Valassi et al. (69)	2012	✅	✅	✅	⚠	❌	❌	✅	❌	High risk
Godbout et al. (63)	2010	✅	✅	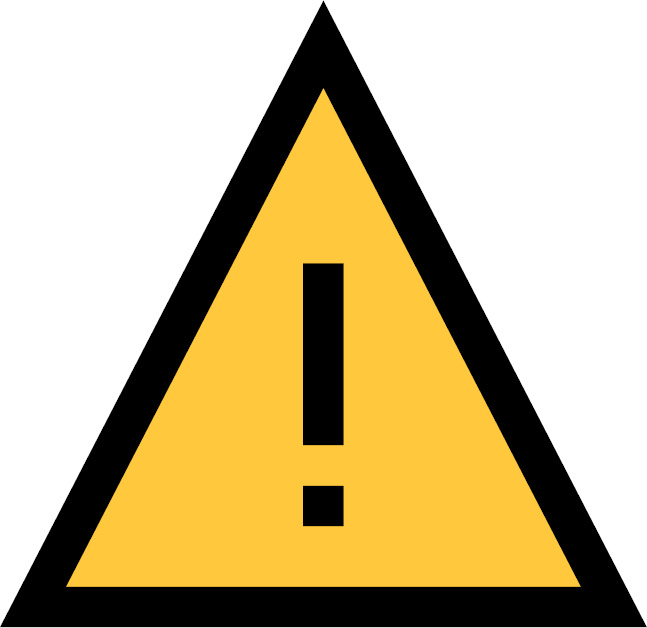	⚠	✅	✅	✅	✅	Some concerns
Ferriere (64)	2016	✅	✅		⚠	✅	✅	✅	✅	Some concerns	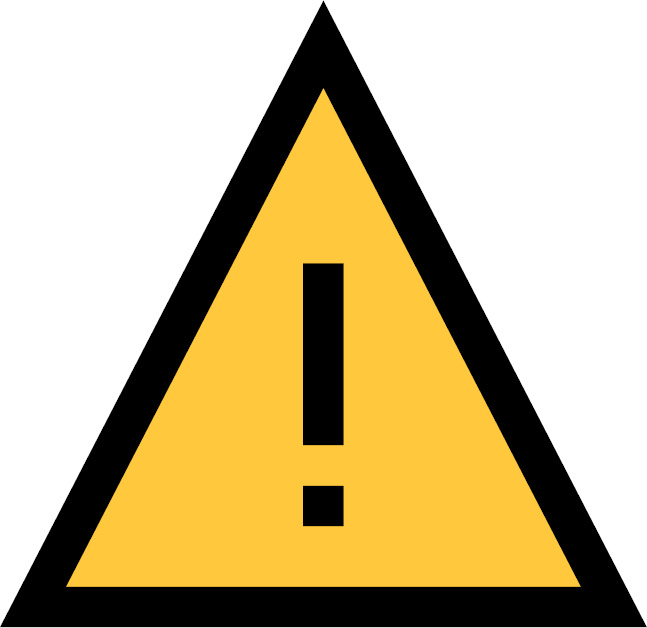
Trementino et al. (59)	2016	✅	✅	✅	✅	❌	❌	✅	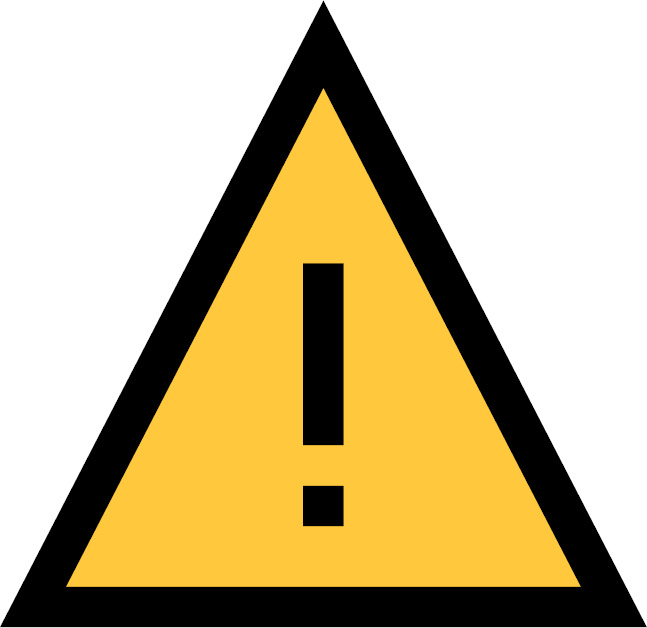	High risk
Luisetto et al. (68)	2001	✅	✅	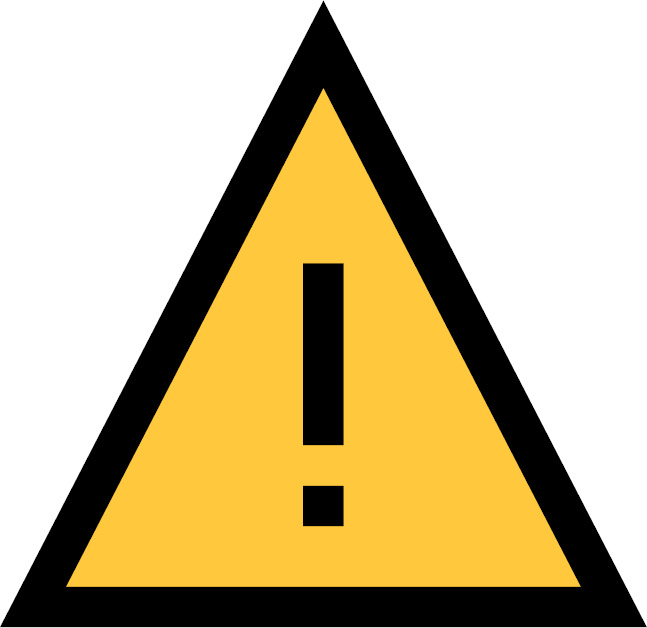	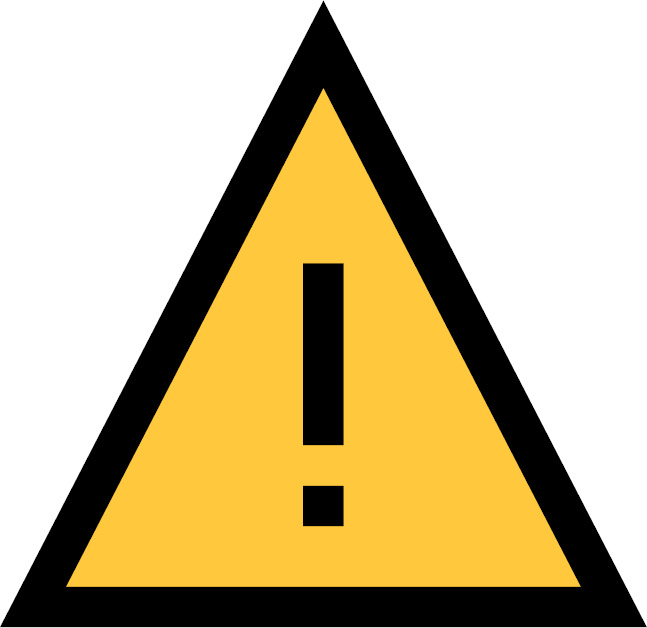	✅	❌	✅	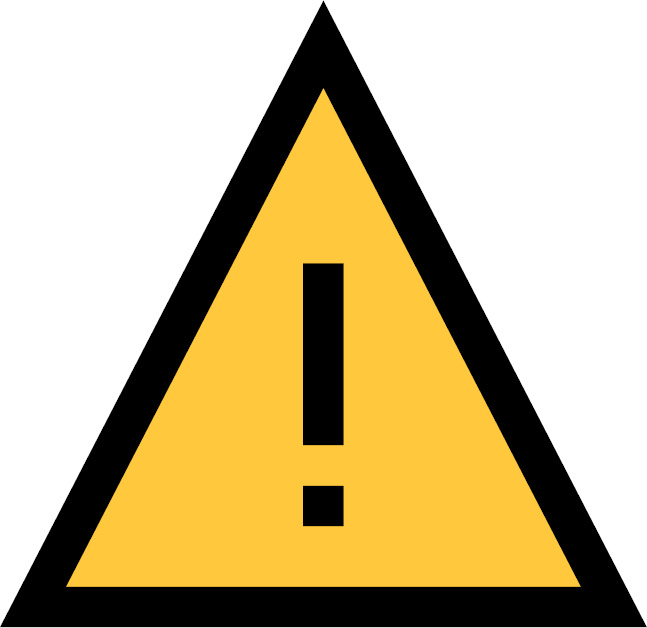	High risk
Ghervan et al. (67)	2015	✅	✅	✅	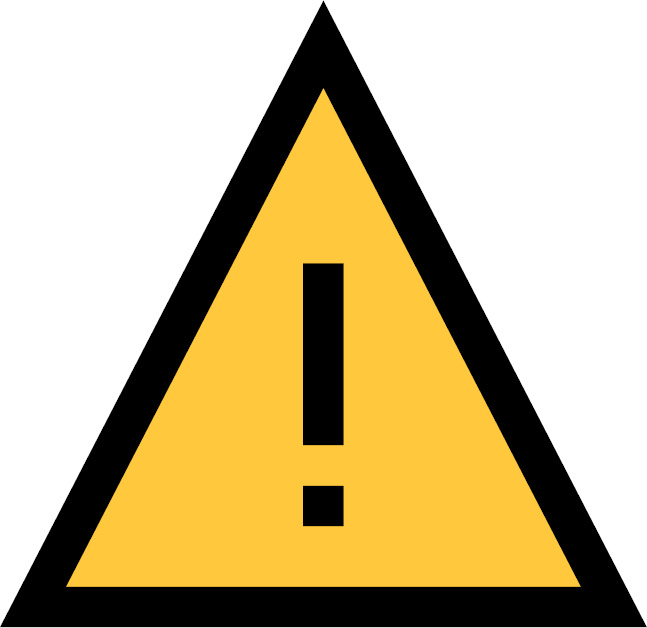	✅	❌	✅	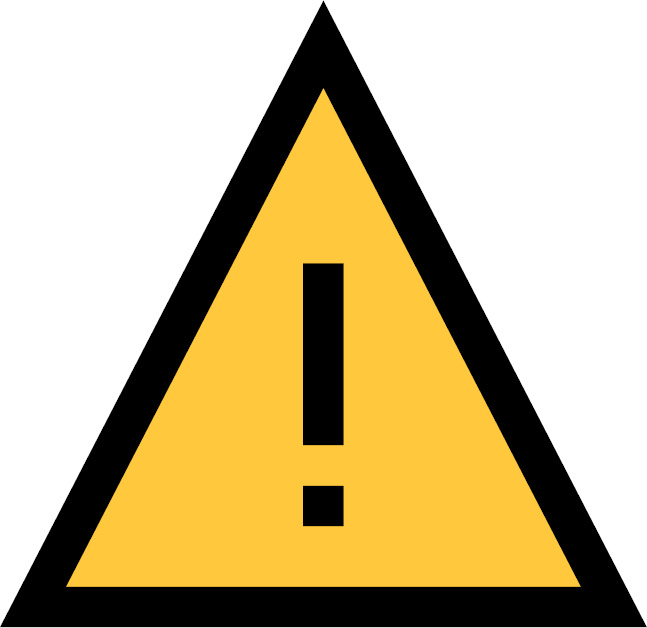	High risk
Ceccato et al. (75)	2018	✅	✅	✅	✅	✅	✅	✅	✅	Low risk
Pivonello et al. (77)	2020	✅	✅	✅	✅	✅	✅	✅	✅	Low risk
Van der Bosch et al. (66)	2014	✅	✅	✅	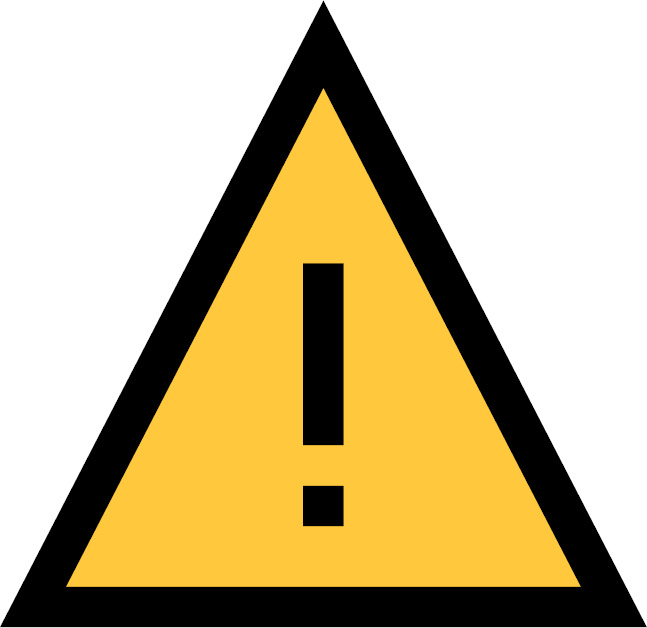	✅	❌	✅	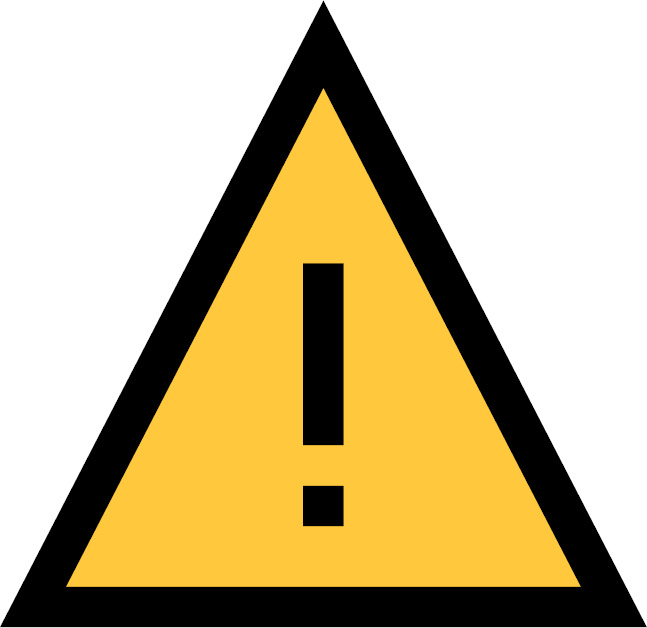	High risk
Moncet et al. (73)	2007	✅	✅	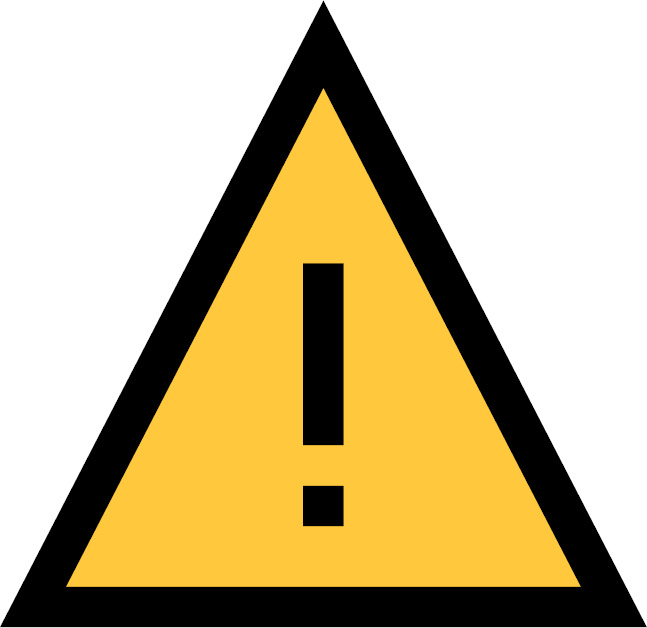	❌	❌	❌	✅	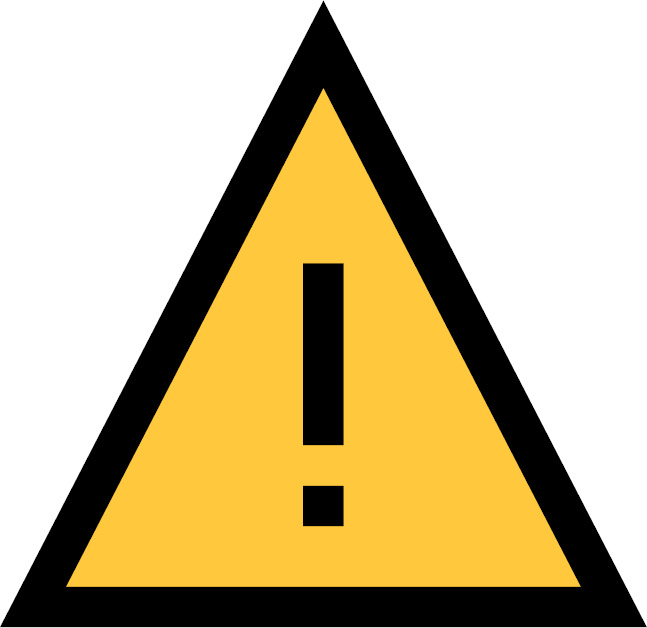	High risk
Sonino et al. (65)	1991	✅	✅	✅	✅	✅	✅	✅	✅	Low risk
Verhelst et al. (74)	1991	✅	✅	❌	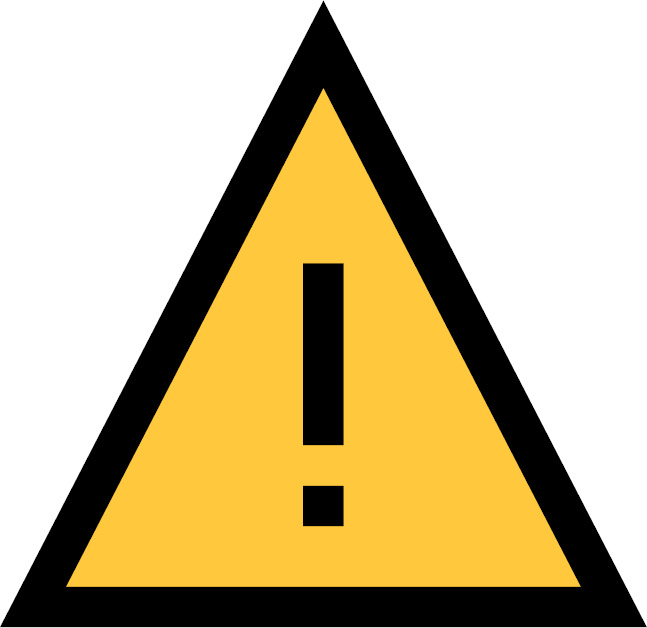	✅	❌	✅	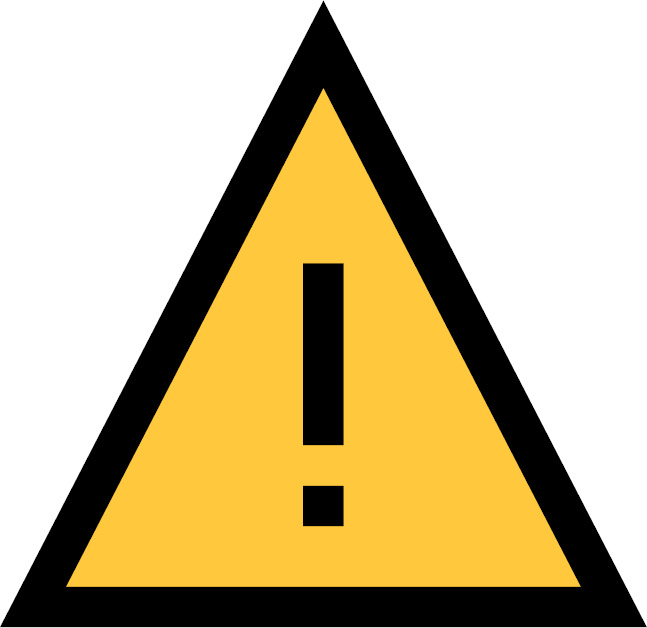	High risk

❌, high risk of bias; ✅, low risk of bias; 
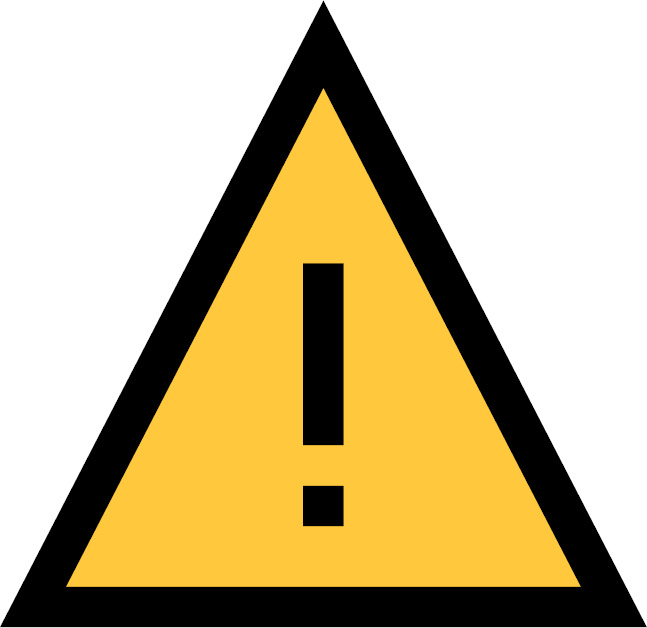
, unclear.

a For overall risk of bias, criteria 4, 5, and 6 were taken into consideration. Overall risk of bias was low if all three were low risk. If one of the three criteria were unclear or high risk, the overall assessment was “some concerns”; if two were unclear or high risk, the overall assessment was “high risk”.

### Proportion of Patients With Disease Control

The treatment effects of pasireotide, cabergoline, ketoconazole, and metyrapone on disease control, as defined by the individual included studies, were pooled in the proportional meta-analyses. Although UFC was most commonly used to measure disease control, Lila et al. used midnight salivary cortisol (MNSC) and low-dose dexamethasone suppression (LDSC) test to define disease control (62), and Daniel et al. used mostly 9 AM cortisol and mean cortisol from a cortisol day-curve (76). The morning serum cortisol was also used by three studies (66, 67, 74).

**Figure 2:**
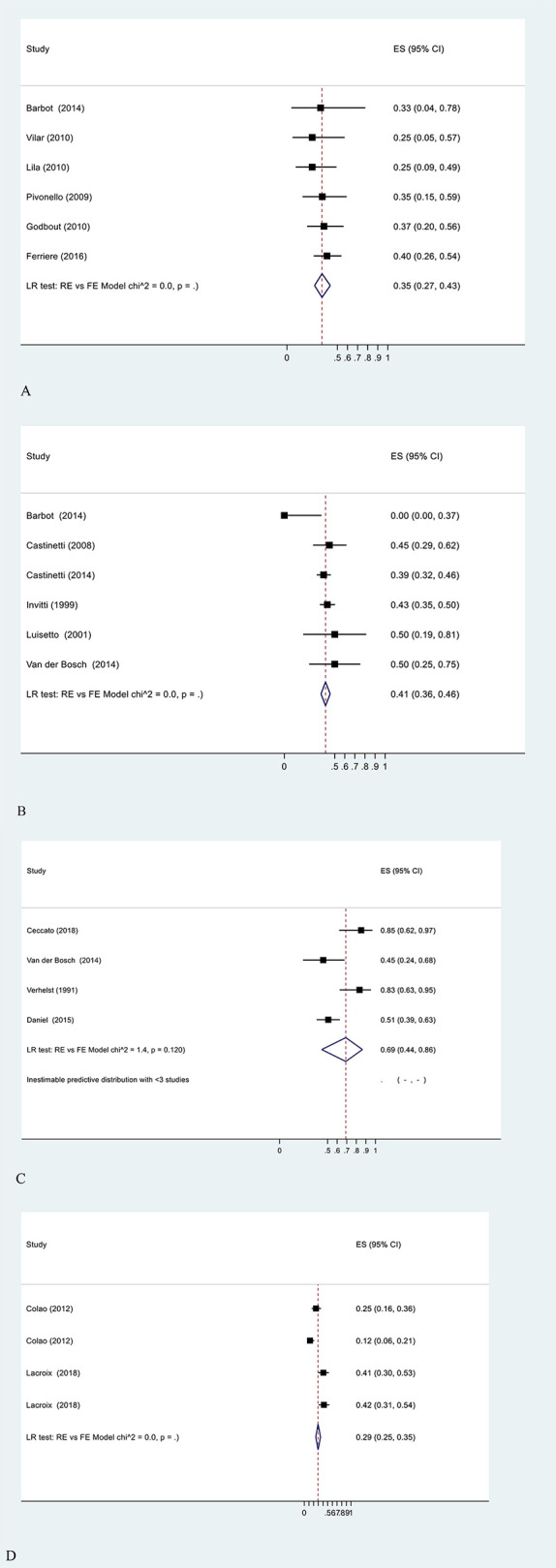
Meta-analysis on the proportions of disease control after treatment with **(A)** cabergoline, **(B)** ketoconazole, **(C)** metyrapone, and **(D)** pasireotide.

**Figure 2** shows the proportional random meta-analysis on disease control. The pooled proportion of control was 35% (27–43%) for cabergoline and 41% (36–46%) for ketoconazole, with low heterogeneity. Although statistic heterogeneity was not confirmed for metyrapone (*p* = 0.12), the small sample of included studies yielded a large CI for`66% of the observed disease control (95%CI: 46–87%, four studies, 66 patients). A subgroup analysis considering studies that considered UFC as the only criteria of disease control was performed for cabergoline (36%, 95% CI: 28–45%, *p* = 0, five studies, 121 patients) and ketoconazole (41%, 95% CI: 36–45%, *P* = 0, 5 studies, 434 patients). With regard to pasireotide, 44% of patients had disease control (30–60%, *χ* ^2^ = 21.3, *p* = 0, PI = 18–74%, eight studies, 522 participants; **Supplementary Figure S1A**). To investigate the heterogeneity, meta-regression was conducted, and it showed that the number of included patients was the variable that explained 50% of heterogeneity. A meta-analysis with the two larger randomized studies showed that the proportion of disease control after pasireotide was 29% (25–35%, *χ* ^2^ = 0, **Figure 2**).

### Improvement of UFC

Because different units of measure were applied to report UFC (*i*.*e*., nmol/24 h, µg/24 h, or number of times above the upper limit of normality), the SMD was used as a measure of effect size to plot pre- and post-intervention data in a meta-analysis. The meta-analysis of studies with pasireotide, cabergoline, and ketoconazole consistently showed a reduction on UFC, although with high heterogeneity. The pooled results for cabergoline, ketoconazole, and pasireotide are shown on **Table 2** (forest plots in **Supplementary Figure S2A**).

**Table 2:** Summary of meta-analysis on the reduction of urinary cortisol pre- and post-intervention.

	SMD	95% CI	*I*²	*P*	PI	Included studies (*n*)	Included patients (*n*)
Pasireotide^a^	-0.94	-1.17, -0.71	51.9%	0.358	-1.60, -0.28	7	503
Randomized studies only	-0.94	-1.14, -0.74	7%	0.028	-1.44, -0.45	2	312
Cabergoline	-2.4	-4.5, -0.25	95%	–	–	4	68
Ketoconazole	-2.88	-5.18, -0.58	96.6%	–	–	4	246

I^2^, Higgins test of heterogeneity; CI, confidence interval; PI, predictive interval; SMD, standard mean deviation.

a Randomized and prospective studies.

The high heterogeneity on the meta-analysis for cabergoline could be explained by one outlier study (12), in which a higher weekly dose (7 mg) was used. The visual analysis of the forest plot for ketoconazole likewise had one outlier study (65), which had a high risk of bias. A sensitivity analysis was performed to explore the heterogeneity for pasireotide. Considering randomized studies only, pasireotide reduced the UFC in -0.94 SD (CI: -1.14, -0.74, *I* ^2^ = 7%) (**Supplementary Figures S2A–C**).

### Improvement of Comorbidities

Pre- and post-intervention data on systolic and diastolic blood pressure, cholesterol, triglycerides, body mass index, and waist circumference were extracted when available. Among these secondary outcomes, improvement of blood pressure (BP) levels was the most commonly reported, although there was variability in reporting and measurement (*i*.*e*., proportion of controlled BP, reduction of the parameter itself). Hence, due to the lack of data and heterogeneity on the reporting of outcomes, a meta-analysis for pasireotide was performed considering the reduction of the parameter itself, disregarding the number of medications, which was poorly described in most studies. **Table 3** shows a metanalysis on the improvement of BMI, waist circumference, and systolic and diastolic BP with pasireotide.

**Table 3:** Summary of meta-analysis on the improvement of comorbidities with pasireotide.

Clinical parameters	SMD	95% CI	*I*²	Included studies (*n*)	Included patients (*n*)
BMI	-1.49	-2.08, -0.90	81.4%	5	381
Randomized studies only	-1.44	-2.07, -0.82	90.5%	2	312
Waist circumference	-3.54	-4.84, -2.24	55%	5	381
Randomized studies only	-3.32	-5.2, -1.43	77%	2	312
Systolic blood pressure	-6.30	-8.46, -4.13	41.8%	7	448
Randomized studies only	-7.18	-10.49, -3.87	51%	2	312
Diastolic blood pressure	-4.32	-5.83, -3.01	0%	6	432
Randomized studies only	-3.95	-5.8, -2.31	0%	2	312

I^2^, Higgins test of heterogeneity; CI, confidence interval; PI, predictive interval; SMD, standard mean deviation.

With regard to cabergoline, Lila et al. observed that four out of 18 patients showed a decrease of 20 mmHg on SBP and 10 mmHg on DBP after 5 months of treatment with cabergoline (62). In another series, a mean reduction on SBP from 141.5 to 118 mmHg after 12 months of follow-up was found (12). Moreover, ketoconazole was also reported to reduce the mean blood pressure from 148/105 mmHg at baseline to 115/85 mmHg after a mean follow-up of 23 months (72). A second study reported that 40% of patients had controlled hypertension after treatment with ketoconazole (71).

### Safety

The proportion of different AE associated with pasireotide, cabergoline, ketoconazole, and metyrapone was also plotted in a proportional meta-analysis, as summarized in **Table 4**. SAEs were reported exclusively for pasireotide, probably because two very low bias randomized studies on pasireotide were included, whereas for other medications, the included studies were mostly prospective or retrospective cohorts.

**Table 4:** Proportional meta-analysis of the frequency of adverse events.

	Frequency of AE	95% CI	Chi-square	*p*	PI	Included studies (*n*)	Included patients (*n*)
Pasireotide							
SAE	0.17	0.04, 0.49	0.6	0.219	–	8	522
Diabetes	0.21	0.15, 0.28	2.1	0.076	0.11, 0.36	8	522
Randomized only	0.25	0.21, 0.30	0	–	–	2	312
Hyperglycemia	0.29	0.15, 0.49	18.4	0	0.06, 0.72	8	522
Randomized only	0.48	0.42, 0.53	0	–	–	2	312
Diarrhea	0.3	0.16, 0.48	17.7	0	0.08, 0.68	5	467
Cholecystitis	0.13	0.02, 0.54	73.2	0	0, 0.92	5	467
Randomized only	0.38	0.33, 0.44	0	–	–	2	312
Nausea	0.21	0.12, 0.33	7.8	0.003	0.06, 0.50	5	467
Randomized only	0.29	0.24, 0.34	0	–	–	2	312
Abdominal pain	0.29	0.14, 0.49	0.8	0.18	–	3	331
Randomized only	0.21	0.16, 0.25	0	–	–	2	312
Headache	0.24	0.19, 0.28	0	–	–	3	331
Randomized only	0.23	0.18, 0.28	0	–	–	2	312
Fatigue	0.2	0.16, 0.25	0	–	–	4	363
Cabergoline							
Escape from treatment	0.14	0.09, 0.21	0	–	–	6	143
Vertigo	0.12	0.07, 0.19	0	–	–	6	143
Nausea	0.1	0.06, 0.16	0	–	–	6	143
Fatigue	0.07	0.03, 0.18	0.1	0.373	–	6	143
Ketoconazole							
Elevated transaminases^a^	0.14	0.11, 0.18	0	–	–	8	366
Diarrhea and/or abdominal pain	0.08	0.04, 0.18	2.6	0.052	–	8	366
Rash	0.03	0.01, 0.09	2.4	0.06	–	8	366
Adrenal insufficiency	0.06	0.04, 0.10	0.2	0.327	–	8	366
Metyrapone							
Nausea	0.18	0.07, 0.40	1.9	0.085	–	4	89
Vertigo	0.17	0.10, 0.26	0	–	–	4	89
Hirsutism	0.17	0.10, 0.26	0	–	–	4	89
Fatigue	0.07	0.01, 0.40	0.1	0.351	–	4	89
Hypokalemia	0.09	0.05, 0.17	0	–	–	4	89

AE, adverse events; CI, confidence interval; Chi-square, heterogeneity; PI, predictive interval; SAE, serious adverse events.

a Includes an increase in alanine aminotransferase and alkaline phosphatase.

In the proportional meta-analyses of main AE associated with pasireotide (*i*.*e*., diabetes, hyperglycemia, cholecystitis, nausea, abdominal pain, and headache), a high heterogeneity was identified. The meta-regression showed that the type of study was the explanatory variable for this heterogeneity, and sensitivity analysis including only randomized trials was performed.

Cabergoline and metyrapone were mainly associated with vertigo and nausea, with low heterogeneity in the meta-analysis. Studies with ketoconazole reported mainly elevated transaminases, rash, and adrenal insufficiency. Diarrhea and/or abdominal pain were assessed as a composed outcome in the meta-analysis.

### Studies Not Included in the Meta-Analyses

No study with temozolomide and levoketoconazole fulfilled our inclusion criteria. Osilodrostat was evaluated in one prospective, open-label, single-arm study with a placebo randomized withdrawal period (77). At 48 weeks, 91 (66.4%, 95% CI: 57.9, 74.3) enrolled patients had a complete response. Sixty-four out of 97 patients (66%) who were treated with osilodrostat throughout the 48 weeks and had a complete response and maintained a complete response for at least 6 months. The most common adverse events included nausea (42%), headache (34%), fatigue (28%), and adrenal insufficiency (28%). Moreover, symptomatic hypocortisolism was reported by 70 (51%) patients, and 58 (42%) patients reported adverse events related to adrenal hormone precursors.

## Discussion

Therapeutic guidelines recommend medical treatment for patients with CD who are not surgical candidates or who have a persistent disease after TSS, although with no preference for either medical treatment (5). Our systematic review set out to assess the effectiveness and safety of medical treatment for CD. The proportional meta-analyses showed a similar proportion of CD control between cabergoline (27–43%), pasireotide (25–35%), and ketoconazole (36–46%). A meta-analysis of metyrapone resulted in 66% of disease control, but with broad CI (46–87%) because most studies were small retrospective cohorts. Moreover, the proportion of disease control with metyrapone may be overestimated because most studies considered the morning serum cortisol as criterion for disease control.

In contrast with other pituitary tumors such as prolactinomas, in which an optimistic response to medical treatment is expected (78), medical treatment for CD induced disease control in less than 50% of patients. Moreover, there are several AE associated with these medications as shown in our meta-analyses. Therefore, new treatment alternatives have been studied, such as osilodrostat and levoketoconazole. A single study on osilodrostat included in our review reported a proportion of disease control of 66% after 48 weeks of follow-up (77). However, osilodrostat is not yet available in most countries, and its safety needs further assessment in larger trials. The only study on levoketoconazole included patients with Cushing’s syndrome of all etiologies and therefore was excluded from our review. This non-controlled study induced disease control in 36% of the 95 patients at the 6-month follow-up (45).

Temozolomide is an orally active alkylating agent that has been used in patients with aggressive corticotroph tumors. Two retrospective case series evaluated temozolomide for patients with aggressive pituitary adenomas and carcinomas (43, 44). Complete remission ocurred in 13% (three out of 23) and 50% (10 out of 20) of the patients. Both studies were excluded from the review because the diagnostic criteria was not clearly reported. Moreover, the outcome measurement for diasease remission was imprecise.

Some limitations of our systematic review must be acknowledged. First, our results were predominantly from uncontrolled studies. Among the controlled studies, Barbot et al. compared cabergoline and ketoconazole (60), while two studies compared two different dosages of pasireotide (10, 54). The only study to compare two different drugs had a high risk of bias due to the incomplete reporting of the randomization process and high uncertainty of the effect size due to the small sample (60). Therefore, lack of controlled studies limits the conclusions with regard to the comparative effectiveness of the medications studied. A second limitation was the low quality of evidence of the included studies, which were mainly small cohorts.

Two similar systematic reviews were published, but with significant differences (14, 79). Gadelha et al. did not perform a meta-analysis due to paucity of the studies included (79). Broersen et al. performed a comprehensive systematic review addressing the medical treatment for Cushing’s syndrome of all etiologies (14). Nevertheless, recently, a large trial on pasireotide was published (54). Therefore, the contribution of our meta-analysis provides an updated overview on the effectiveness of the medical treatment for CD. In addition to disease control, this is the first study to pool the effect on UFC reduction, comorbidities, and AE.

Disease control was defined by most of the included studies as UFC below the upper limit of normality, with few exceptions. Lila et al. considered MNSC and LDSC, while four studies considered the morning serum cortisol (66, 67, 74, 76). Among the four studies included in the proportional meta-analyses of disease control of metyrapone, only one considered UFC as a criterion for disease control (75). There is a good correlation between the normalization of UFC and the improvement of signs and symptoms of hypercortisolism (32). Moreover, the normalization of UFC is associated with a low recurrence risk, and therefore some studies advocate that it should be considered as the main criterion to determine control (3).

Despite being not within the scope of this review, a combination of drugs has shown promising results for CD. In the second phase of the randomized study by Barbot et al., the combination of cabergoline and ketoconazole achieved UFC normalization in 79% of patients and a significant improvement in the symptoms of hypercortisolism. These results persisted for at least 6 months, with a few adverse events (60). Feelders et al. treated 17 patients first with pasireotide as monotherapy, then combined pasireotide with cabergoline, and then added ketoconazole if the patients did not achieve control (52).

The small sample, however, limits the conclusion with regard to the effectiveness of the combined treatment. A phase II, open-label, multicenter clinical trial on the combination of pasireotide and cabergoline, the CAPACITY study, will assess the efficacy and safety in CD patients (80).

In conclusion, medical treatment is a valid treatment alternative for patients who had a recurred hypercortisolism after TSS or who had contraindications for surgery. The proportion of disease control after treatment with cabergoline, ketoconazole, and pasireotide identified in our meta-analysis may support the clinical decision. New therapeutic options should be investigated due to the limited efficacy and tolerability of the currently available medical treatment for patients with Cushing’s disease.

## Practical Implications

Pasireotide is approved for CD within the Brazilian Unified Health System, while cabergoline and ketoconazole are used as off-label medications. These results may support the inclusion of cabergoline and ketoconazole as alternative second-line treatment for patients with CD in Brazil as well as in other countries.

## Systematic Review Registration

This systematic review was registered in the International Prospective Register of Systematic Reviews (PROSPERO CRD42020205567).

## Data Availability Statement

The original contributions presented in the study are included in the article/**Supplementary Material**. Further inquiries can be directed to the corresponding author.

## Author Contributions

VS and CB conceptualized and designed the study. JS and MD developed the search strategies. VS and JS independently screened eligible studies. JS and AN extracted data from the included studies and assessed the individual risk of bias. VS and JS assessed in pairs and independently the risk of bias. VS performed the meta-analysis. VS supervised all the phases of this review and refereed any disagreement to avoid errors. All authors contributed to the article and approved the submitted version.

## Conflict of Interest

The authors declare that the research was conducted in the absence of any commercial or financial relationships that could be construed as a potential conflict of interest.

## Publisher’s Note

All claims expressed in this article are solely those of the authors and do not necessarily represent those of their affiliated organizations, or those of the publisher, the editors and the reviewers. Any product that may be evaluated in this article, or claim that may be made by its manufacturer, is not guaranteed or endorsed by the publisher.
