# Supplementary material for: Effectiveness of Medical Treatment of Cushing’s Disease: A Systematic Review and Meta-Analysis
Source: Front Endocrinol (Lausanne). 2021 Sep 17;12:732240. doi: 10.3389/fendo.2021.732240 (PMC8485729; doi:10.3389/fendo.2021.732240)
Supplement: Supplementary file 1 [file DataSheet_1.docx]

***Supplementary Material***

**Effectiveness of Medical treatment of Cushing’s disease: systematic review and meta-analysis**

Simoes Correa Galendi, J^1^; Correa Neto, ANS^2^, Demetres, M^3^ , Boguszewski CL^4^, Nunes-Nogueira, VS^5^

Contents

Table 1A. Search Strategy Pubmed – Searched on August, 20th 2020

Table 2A. Search Strategy Pubmed – Searched on January, 16th 2021

Table 3A. Characteristics of included studies

Figures

Figure 1A Proportional meta-analysis on disease control after treatment with pasireotide.

Figure 2A Meta-analysis on reduction of urinary cortisol after treatment with (A) Cabergoline, (B) Ketoconazole and (C)Pasireotide.

Figure 3 Subgroup meta-analysis on proportion of disease control after treatment with (A) Cabergoline and (B) Ketoconazole, considering UFC as only outcome measure.

*Table 1A Search Strategy PubMed – Searched on August, 20^th^ 2020*

| #1 | ("Pituitary ACTH Hypersecretion"[Mesh] OR Pituitary ACTH Hypersecretion OR Pituitary Adrenocorticotropic Hormone Hypersecretion OR Cushing's OR Cushing OR Cushings OR Pituitary-Dependant Hypercortisolism OR Pituitary-Dependent Hypercortisolism OR Pituitary-Dependant Hypercortisolisms OR Pituitary-Dependent Hypercortisolisms OR corticotropin induced adrenocortical hyperplasia OR corticotropinoma OR corticotropinomas OR "ACTH-Secreting Pituitary Adenoma"[Mesh] OR ACTH-Secreting Pituitary Adenoma OR ACTH-Secreting Pituitary Adenomas OR Adrenocorticotropic Hormone-Secreting Pituitary Adenoma OR Adrenocorticotropic Hormone-Secreting Pituitary Adenomas OR Corticotroph Adenoma OR Corticotroph Adenomas OR Pituitary Corticotropin-Secreting Adenoma OR Pituitary Corticotropin-Secreting Adenomas OR ACTH-Producing Pituitary Adenoma OR Adrenocorticotropic Hormone-Producing Adenoma OR Adrenocorticotropic Hormone-Producing Adenomas OR ACTH) | 76,685 |
| --- | --- | --- |
| #2 | ("pasireotide" [Supplementary Concept] OR SOM-230 OR SOM230 OR SOM 230 OR Pasireotide OR signifor) | 609 |
| #3 | ("Ketoconazole"[Mesh] OR Ketoconazole OR R-41400 OR R 41400 OR R41400 OR R41 400 OR Nizoral OR akorazol OR anfuhex OR antanazol OR beatoconazole OR bigazol OR cetonaxOR comozol OR conazol OR cremosan OR daktagold OR dezoral OR dio 902 OR dio902 OR extina OR formyco OR fugen OR funazole tabs OR funet OR fungarest OR fungaway OR fungazol tabs OR fungicide tabs OR fungiderm-k OR funginoc OR funginox tabs OR fungoral OR kenazol OR kenazole OR kesnazol OR ketazol OR keto-comp OR keto-crema OR keto-shampoo OR ketocanazole OR ketoconazol OR ketoderm OR ketoisdin OR ketomed OR ketomicin OR ketomicol OR ketona OR ketozal OR ketozol OR ketozole OR kezon OR konaturil OR kw 1414 OR larry OR lusanoc OR micoral OR mizole OR mizoron OR mycofebrin OR nastil OR nazole OR neutrogena t/sal OR nisoral OR niz creme OR niz shampoo OR oxocanazole OR oxoconazole OR oxonazol OR panfungol OR pasalen OR picamic OR prenalon OR pristine (drug) OR pristinex OR profungal OR r 41, 400 OR r 41400 OR sebizole OR sporium OR sporoxyl OR sporozol OR termizol OR terzolin OR triatop lotion OR xolegel OR zoralin tabs OR zorinax) | 15,185 |
| #4 | ("cabergoline" [Supplementary Concept] OR cabergoline OR Galastop OR FCE 21336 OR FCE-21336 OR Cabaser OR Cabaseril OR Dostinex OR cabergoline diphosphate OR cabergolin OR cabergolina OR cabest OR actualene OR cabarsuss OR sogilen OR sostilar OR velactis) | 1,777 |
| #6 | (#2 OR #3 OR #4) | 17,430 |
| #7 | (#1 AND #6) | 719 |

*Table 2A Seacrh Startegy PubMed – Searched on January, 16^th^ 2021*

| #1 | ("Pituitary ACTH Hypersecretion"[Mesh] OR Pituitary ACTH Hypersecretion OR Pituitary Adrenocorticotropic Hormone Hypersecretion OR Cushing's OR Cushing OR Cushings OR Pituitary-Dependant Hypercortisolism OR Pituitary-Dependent Hypercortisolism OR Pituitary-Dependant Hypercortisolisms OR Pituitary-Dependent Hypercortisolisms OR corticotropin induced adrenocortical hyperplasia OR corticotropinoma OR corticotropinomas OR "ACTH-Secreting Pituitary Adenoma"[Mesh] OR ACTH-Secreting Pituitary Adenoma OR ACTH-Secreting Pituitary Adenomas OR Adrenocorticotropic Hormone-Secreting Pituitary Adenoma OR Adrenocorticotropic Hormone-Secreting Pituitary Adenomas OR Corticotroph Adenoma OR Corticotroph Adenomas OR Pituitary Corticotropin-Secreting Adenoma OR Pituitary Corticotropin-Secreting Adenomas OR ACTH-Producing Pituitary Adenoma OR Adrenocorticotropic Hormone-Producing Adenoma OR Adrenocorticotropic Hormone-Producing Adenomas OR ACTH) | 77,396 |
| --- | --- | --- |
| #2 | "Osilodrostat" [Supplementary Concept] OR (benzonitrile, 4-((5R)-6,7-dihydro-5H-pyrrolo(1,2-c)imidazol-5-yl)-3-fluoro-) OR (4-((5R)-6,7-dihydro-5H-pyrrolo(1,2-c)imidazol-5-yl)-3-fluoro-benzonitrile) OR ((+)-Osilodrostat) OR (Isturisa) OR (LCI699) | 49 |
| #3 | (Recorlev) OR (LEVOKETOCONAZOLE) OR ((2S,4R)-ketoconazole) OR (NormoCort COR-003) | 20 |
| #4 | "Temozolomide"[Mesh] OR (Methazolastone) OR (Temodal) OR  (Temodar) OR (Temozolomide Hexyl Ester) | 8,304 |
| #5 | "Metyrapone"[Mesh] OR (Methbipyranone) OR (Methopyrapone) OR (Metopirone) | 4,610 |
| #6 | (#2 OR #3 OR #4 OR #5) | 12,961 |
| #7 | (#1 AND #6) | 830 |

*Table 3A Characteristics of included studies*

| **Included studies** | | **Population** | | | | **Intervention** | **Outcomes** | | | **Follow up** |
| --- | --- | --- | --- | --- | --- | --- | --- | --- | --- | --- |
| Author year | Study type | N | Mean age  (range or SD) | F/M | TSS | Dosage | Criteria for disease control | AE reported | Comorbidities |  |
| **Pasireotide** | | | | | | | | | | |
| Colao 2012 | Randomized | 80 | 40 (18-67) | 62/18 | 64 | 900 mcg | UFC (nmol/24h) | Yes | DM, SBP, DBP, Cholestherol, TG, BMI, WC | 6 and 12 months |
|  |  | 76 | 40 (19-71) | 64/12 | 64 | 600 mcg |  |  |  |  |
| Lacroix 2018 | Randomized | 76 | 38,6 (13) | 58/18 | 59 | 30 mg LAR | UFC  (xULN) | Yes | DM, SBP, DBP, Cholestherol, TG, BMI, WC | 7 and 12 months |
|  |  | 76 | 38,3 (12,5) | 60/16 | 64 | 10 mg LAR |  |  |  |  |
| Albani 2018 | Prospective | 16 | 43,8 (12,1) | 11/5 | 16 | 1800 mcg | UFC  (xULN) | Yes | DM, SBP, DBP, Cholesterol, TG, BMI, WC | 6 and 12 months |
| Barbot 2018 | Prospective | 21 | 46 (12,2) | 16/5 | 20 | 600 mcg | UFC  (xULN) | No | DM, SBP, DBP, Cholesterol, TG, BMI, WC | 12 months |
| Boscaro 2014 | Prospective | 18 | 43(22-73) | 17/2 | - | 600 mcg | UFC (nmol/24h) | Yes | DM, SBP, DBP | 6 months |
| Fleseriu 2018 | Prospective | 104 | 45,5(13,1) | 84/20 | 84 | 600/900mcg | UFC nmol/24h | Yes | DM, SBP, DBP | 24 weeks |
| Pivonello 2019 | Prospective | 32 | 47 (21-71) | 25/7 | - | 600 mcg | UFC mcg/24h | Yes | DM, SBP, DBP, Cholesterol, TG, BMI, WC | 6 months |
| Trementino 2016 | Retrospective | 19 | 49,6 (15,7) | 12/7 | 19 | 600 mcg | UFC  xULN) | Yes | Nort reported | 6 months |
| **Cabergoline** | | | | | | | | | | |
| Barbot 2014 | Randomized | 6 | 52 (37-65) | 6/0 | 6 | 3 mg/week | UFC (nmol/24h) | Yes | DM, Mean BP, BMI | 6 months |
| Vilar 2010 | Prospective | 12 | 42,8 (6,2) | 8/4 | 12 | 2-3 mg/week | UFC (mcg/24h) | Yes | Not reported | 6 months |
| Lila 2010 | Prospective | 18 | 27,4 (13-48) | 10/8 | 18 | 3.6 mg/week | MNSC and LDSC | No | Nor reported | 12 months |
| Pivonello 2009 | Prospective | 20 | 39,9 (26-60) |  | 20 | 1-7mg/week | UFC (mcg/24h) | Yes | DM, SBP, DBP, BMI | 12 months |
| Godbout 2010 | Retrospective | 30 | 39 (12) | 25/5 | 27 | 6mg/week | UFC (nmol/24h) | Yes | Not reported | 6 months |
| Ferriere 2017 | Retrospective | 53 | 37,5 (7-78) | 46/7 | 44 | 1mg/week (mean) | UFC  (xULN) | Yes | DM, SBP, DBP, BMI | 7 months |
| **Ketoconazole** | | | | | | | | | | |
| Barbot 2014 | Randomized | 8 | 52 (39-72) | 6/2 | 4 | 200mg/day | UFC (nmol/24h) | Yes | WC, BMI | 6 months |
| Ghervan 2015 | Prospective | 12 | 35,9 | 5/7 | 3 | 300-800mg/day | Morning serum cortisol | Yes | SBP, DBP , Cholesterol, TG, BMI | 15 months |
| Luisetto 2001 | Prospective | 10 | 45,1 (7,1) | 1/9 | 10 | 300-600mg/day | UFC (nmol/24h) | No | Not reported | 44 months |
| Moncet 2007 | Prospective | 52 | 37 (33-46) | 10/42 | 8 | 600mg/day | UFC (nmol/24h) | No | Not reported | 9 months |
| Sonino 1991 | Prospective | 28 | 38,5 (14-67) | 4/24 | 0 | 400-800mg/day | UFC (nmol/24h) | Yes | Not reported | 6 months |
| Castinetti 2008 | Retrospective | 38 | 47,1 (26-73) | 28/5 | 17 | 600-1200mg/day | UFC (nmol/24h) | Yes | DM, SBP, DBP | 23 months |
| Castinetti 2014 | Retrospective | 200 | 41,9 (15,8) | 156/44 | 144 | 200-1200mg/day | UFC  (xULN) | Yes | Not reported | 33 months |
| Valassi 2012 | Retrospective | 17 | 47 (13) | 54/8 | - | Not described | UFC | Yes | Not reported | 6 months |
| Invitti 1999 | Retrospective | 178 | 36 (11-72) | 239/49 | - | not described | UFC | No | Not reported | 115 months |
| Van der Bosch 2014 | Retrospective | 16 | 50 (16-65) | 4/12 | 0 | 720mg/day | UFC (nmol/24h) or morning serum cortisol | Yes | Not reported | 6 months |
| **Metyrapone** | | | | | | | | | | |
| Ceccato 2018 | Prospective | 20 | 45 (3) | 5/15 | 14 | 750mg/day | UFC (nmol/24h) | Yes | SBP, DBP, BMI, WC | 24 months |
| Valassi 2012 | Retrospective | 23 | 41 7–70) | - | 0 | 750-1000mg/day | UFC (nmol/24h) | Yes | Not reported | 4 months |
| Van der Bosch 2014 | Retrospective | 22 | 50 (16-65) | 6/16 | 0 | 2477mg/day | UFC (nmol/24h) or morning serum cortisol | Yes | Not reported | 6 months |
| Verhelst 1991 | Retrospective | 24 | 57 (9-68) | 12/12 | 2 | 500mg/day | Morning serum cortisol | Yes | Not reported | 27 months |
| Daniel 2015* | Retrospective | 71 | 45.2 | - | 21 | 939.2 mg/day-1432 mg/day | UFC (nmol/24h) or morning serum cortisol or cortisol day-curves | No | Not reported | 18 months |
| **Osilodrostat** | | | | | | | | | | |
| Pivonello 2020 | Randomized | 36 | Median: 41 (IQR: 37.5 - 51.5) | 6/30 | 32 | 10mg/day | UFC (nmol/24h) | No | Fasting blood glucose, HbA1C, SBP, DBP, cholesterol, bodyweight | 12 months |

**Abbreviations: TSS: number of patients with prior TSS transsphenoidal surgery; F/M: proportion of female/male; LAR: long action release; UFC: Urinary Free Cortisol; MNSC: Midnight salivary cortisol; LDSC: and low dose dexamethasone suppression cortisol test; BMI: Body Mass Index; xULN: times the upper limit of normality. *Data provided by the authors considering pituitary etiology only.**

*Figure 1A Proportional meta-analysis on disease remission after treatment with pasireotide.*

A

B

C

Abbreviations: SMD: Standard mean difference; CI: Confidence interval.

Figure 2A Meta-analysis on reduction of urinary cortisol after treatment with (A) Cabergoline, (B) Ketoconazole and (C)Pasireotide.

A

B

*Figure 3 Subgroup meta-analysis on proportion of disease control after treatment with (A) Cabergoline and (B) Ketoconazole, considering UFC as only outcome measure.*
